# Effective doses of remimazolam for sedation in paediatric magnetic resonance imaging following dexmedetomidine premedication: a dose-finding study

**DOI:** 10.1186/s12871-026-03848-2

**Published:** 2026-04-20

**Authors:** Wanjun Zheng, Yanhua Cui, Yu Gao, Jiaxin Dong, Zhipeng Zhong, Bilian Li

**Affiliations:** Department of Anaesthesiology, Guangzhou Women and Children’s Medical centre, Guangdong Provincial Clinical Research centre for Child Health, Guangzhou Medical University, Guangzhou, China

**Keywords:** Remimazolam, Dexmedetomidine, Magnetic resonance imaging, Paediatric, Sequential sedation regimen

## Abstract

**Background:**

Effective sedation for paediatric magnetic resonance imaging (MRI) requires balancing depth control and safety. This dose-finding study aims to establish the 50% (ED_50_) and 90% (ED_90_) effective doses of intravenous remimazolam following intranasal dexmedetomidine premedication to optimise this sequential regimen.

**Methods:**

In this single-centre trial (March-December 2024), children aged 3 months to 6 years requiring MRI sedation were enrolled. After intranasal dexmedetomidine (3 µg·kg⁻¹), children adequately sedated by dexmedetomidine alone were excluded. 150 children with inadequate sedation were stratified by age (3–12 months, 1–3 years, 3–6 years) and administered remimazolam via Dixon’s up-and-down method. ED_50_ and ED_90_ with 95%CI were calculated via probit regression. Vital signs, adverse events, awake time, and post-sedation side effects were recorded.

**Results:**

Remimazolam for successful sedation maintained consistent ED_50_ values of 0.12 mg·kg^− 1^ across all paediatric age groups. ED_90_ values slightly increased from 0.20 mg·kg^− 1^ in infants and toddlers to 0.23 mg·kg^− 1^ in preschoolers. MRI was successfully completed in 93.3% of patients, while 6.7% required additional medication during the examination. Adverse events occurred in 15.3% of children, including transient hypotension in 2%, bradycardia in 0.7%, and hypoxia in 0.7%; all events were self-limiting without intervention.

**Conclusions:**

This prospective study defines the ED_50_ and ED_90_ of remimazolam following dexmedetomidine premedication in dexmedetomidine-non-responders for paediatric MRI sedation. The sequential regimen demonstrated consistent weight-based dosing requirements with a favorable safety profile.

**Trial registration:**

The trial was prospectively registered in the Chinese Clinical Trial Registry (chictr.org.cn; No. ChiCTR2400081218) on February 26, 2024.

**Supplementary Information:**

The online version contains supplementary material available at 10.1186/s12871-026-03848-2.

## Introduction

The expanding use of magnetic resonance imaging (MRI) in paediatric populations presents unique clinical challenges, as prolonged scan durations and high noise levels frequently lead to motion artifacts. Failed examinations often require repeated sedation, increasing healthcare costs and risks to children. This underscores the need for effective and safe sedation alternatives. While propofol is widely utilized in paediatric anaesthesia with a well-documented safety profile, particularly in total intravenous anaesthesia and critical care settings [1, 2], some studies have noted potential adverse effects such as respiratory depression and hemodynamic instability, which may be more relevant in young children due to physiological immaturity [3–5]. This has driven interest in alternative sedation regimens that could further mitigate these risks.

Intranasal dexmedetomidine has gained popularity as a non-invasive option for paediatric premedication, offering easy administration, minimal respiratory depression, and physiological sleep-like states compared to traditional sedatives [6]. While intravenous dexmedetomidine demonstrates high efficacy as a sole sedative for paediatric MRI, the inherently loud and stimulating nature of MRI scanning may disrupt sedation with intranasal dexmedetomidine alone [7]. This is reflected in studies reporting variable success rates for intranasal dexmedetomidine monotherapy in MRI, ranging from 30% to 70%, along with high scan interruption rates and a significant need for rescue medication [7, 8]. Furthermore, Ibrahim et al. reported a 20.6% failure rate to complete MRI even with a combination regimen of intranasal dexmedetomidine (3 µg·kg^− 1^) and midazolam [9]. Therefore, while effective monotherapy protocols exist, particularly for intravenous administration, optimizing sedation strategies remains necessary. Combination regimens of dexmedetomidine with other sedatives may offer benefits by refining dosing, potentially enhancing safety and efficacy, and improving success rates for paediatric MRI sedation [2, 10], necessitating dedicated dose-finding studies.

Remimazolam is a newly developed benzodiazepine derivative characterized by its ultra-short-acting properties. Its metabolism primarily relies on plasma esterases, thereby minimizing hepatic and renal metabolic demands, and it achieves rapid sedative effects [11, 12]. Phase III trials in adults have shown that remimazolam provides better haemodynamic and respiratory stability than propofol, as reflected by fewer cases of decreased blood pressure and oxygen desaturation [13]. Growing interest in paediatric remimazolam applications has driven clinical trials evaluating its safety and efficacy, with an emphasis on its pharmacokinetics, dosing regimens, and postoperative adverse reaction prevention [14–17]. Emerging evidence supports remimazolam as an alternative to propofol for paediatric anaesthesia, with comparable maintenance success rates and significantly lower adverse events in recent trials [18]. However, despite an editorial review hypothesizes remimazolam as a promising MRI sedative medication [19], no clinical evidence exists to support its use in paediatric MRI.

To address this gap, we designed a sequential regimen employing intranasal dexmedetomidine followed by intravenous remimazolam for paediatric MRI procedures. We hypothesize this combination may yield synergistic effects by first inducing physiological sleep with dexmedetomidine then maintaining motionless sedation with remimazolam, while minimizing cardiorespiratory risks. The 30-minute interval between dexmedetomidine administration and remimazolam injection was designed to match the peak effect of dexmedetomidine, aiming to achieve potential synergistic effects [20]. This study aims to determine the 50% (ED_50_) and 90% (ED_90_) effective doses of remimazolam after dexmedetomidine premedication in children aged 3 months-6 years, while systematically evaluating the safety and efficacy of this sequential pharmacological regimen.

## Methods

This study was approved by the Guangzhou Women and Children’s Medical centre Ethics Committee (Approval Code: 2024-045A01) and written informed consent was obtained from all subjects’ guardian participating in the trial. Off-label paediatric application of remimazolam was approved by the institutional Committee on Pharmaceutical Affairs.

This prospective study enrolled children between 3 months and 6 years of age classified as ASA I-III and scheduled for MRI examinations lasting ≤ 60 min. ASA III inclusion criteria: Stable systemic disease (e.g., controlled epilepsy). Patient recruitment occurred between March and December 2024. The exclusion criteria were as follows: haemodynamic instability; severe cardiovascular, hepatic, or renal function impairment; active upper respiratory infection; moderate to severe obstructive sleep apnoea syndrome (OSA) or other airway obstructive diseases; known hypersensitivity to dexmedetomidine or remimazolam; and patients having received any other sedative 48 h before sedation. After enrollment, children achieving successful sedation with dexmedetomidine monotherapy were excluded from the remimazolam dose-finding analysis.

An anaesthesiologist measured the sedation level based on the University of Michigan Sedation Scale (UMSS, Table 1). Sedation success was defined as maintaining UMSS ≥ 2 without gross movement throughout the initial 2-minute MRI sequences and subsequently completing the scan without supplemental sedative doses. Children failing to meet either criterion were classified as sedation failures.

**Table 1:** Sedation and behaviour assessment scales

University of Michigan Sedation Scale (UMSS)	
0	Awake or Alert
1	Minimally sedated: tired/sleepy, appropriately responds to verbal conversation and/or sounds
2	Moderately sedated: somnolent/sleeping, easily aroused with light tactile stimulation
3	Deeply sedated: deep sleep, arousable only with significant physical stimulation
4	Unarousable
Behaviour Assessment Scores	
1	Calm and cooperative
2	Anxious but reassurable
3	Anxious and not reassurable
4	Crying or resisting

During the routine pre-sedation visit, an anaesthesiologist collected demographic data, medical history, and sleep deprivation duration. After establishing intravenous access, children were measured pulse rate (PR), non-invasive blood pressure (NBP) and oxygen saturation (SpO_2_) at baseline. Following the drying and cleaning of the nasal passages, children received dexmedetomidine hydrochloride injection (3 µg·kg^− 1^, 100 µg·ml^− 1^; Jiangsu Hengrui Pharma Corporation, Lianyungang, China) intranasally, with half in each nostril. Patients remained in a supine position for two minutes to optimise drug absorption. At 30 min post-administration, children with sedation failure were included in the study and subsequently administered remimazolam intravenously; those achieving adequate sedation at 30 min were excluded regardless of subsequent MRI completion status.

Children were prospectively stratified by age into three groups based on WHO child development stages to account for maturation-related pharmacokinetic variations: infants (3–12 months), toddlers (1–3 years), and preschoolers (3–6 years). Within each stratum, children were consecutively enrolled until a predefined sample size of 50 participants per group was reached. All participants subsequently received intravenous remimazolam.

Before the examination, a predetermined dose of remimazolam tosilate (36 mg, Jiangsu Hengrui Pharma Corporation, Lianyungang, China) was reconstituted in 0.9% sterile saline solution to a concentration of 1 mg·ml^− 1^ and then administered intravenously in about 30 s at a constant speed. The preparation and administration of the study drug were performed by two anaesthesiologists who were not involved in any subsequent assessments of the children. They had no contact with assessors and were prohibited from disclosing dosing information. Assessors responsible for evaluating sedation efficacy, adverse events, and recovery outcomes remained blinded to dosing details through physical separation during procedures, standardized data collection forms that excluded dosing information, and enforced non-communication between administration and assessment teams until final analysis.

The sequential dose was assigned using Dixon’s up-and-down method [21]. The initial remimazolam dose in three age groups was 0.2 mg·kg^− 1^, with a step size of 0.05 mg·kg^− 1^. The dosage adjustment was determined by the previous patient’s response: for sedation failure, the remimazolam dose was raised by 0.05 mg·kg^− 1^ in the next child; for success, it was reduced by 0.05 mg·kg^− 1^. If adequate sedation was not attained within 1 min post-injection, supplementary remimazolam doses (0.1 mg·kg^− 1^) were repeated until success or reaching the maximum pre-MRI cumulative dose (0.5 mg·kg^− 1^). Propofol 0.5 mg·kg^− 1^ served as rescue medication if sedation failure persisted after reaching this threshold. Intermittent supplementation with 0.1 mg·kg^− 1^ remimazolam was provided when sedation proved inadequate during the procedure.

Vital signs (PR, NBP and SpO_2_) and UMSS were recorded at baseline, 1 min after each intravenous administration, upon MRI completion, at awakening, and at 10-min intervals throughout. Hypotension was defined as systolic blood pressure (SBP) ≥ 20% below age-specific norms and bradycardia as HR ≥ 20% below age-specific norms [22]. Dopamine was administered intravenously for hypotension, and atropine for bradycardia, as clinically indicated. No supplemental oxygen was routinely administered during the study. Hypoxia was defined as SpO_2_ below 93% in patients without supplementary oxygen. Behaviour responses to drug delivery were assessed using a four-point behaviour scale (Table 1). A Siemens Skyra 3.0 Tesla scanner (Siemens, Erlangen, Germany) was employed for MRI scanning, generating an average noise output of approximately 88 dB. All children undergoing MRI received pediatric-sized earmuffs for hearing protection prior to scanning.

After MRI, parents or legal guardians were encouraged to use tactile stimulation to facilitate the child’s emergence from sedation. Awake time, defined as the period between successful sedation and the child’s UMSS returning to 0 or 1 after completing the examination, was systematically recorded along with immediate adverse effects. Discharge criteria required fulfilment of both UMSS ≤ 1 and Aldrete score ≥ 9. After discharge, parents were instructed to monitor and document post-sedation outcomes, including delayed adverse effects (e.g., nausea and vomiting, respiratory depression, agitation) and time to resume age-appropriate activities. This information was obtained through a telephone interview conducted at 24 and 48 h post-sedation.

The primary outcomes of this study were the ED_50_ and ED_90_ of remimazolam required to achieve successful sedation for MRI examination. Secondary outcomes were: (1) sedation-related parameters (cumulative remimazolam dose, supplementary dosing needs, behaviour scores during dexmedetomidine and remimazolam administration); (2) procedural metrics (examination time); (3) safety assessments (adverse events rate); (4) functional recovery metrics (awake time, immediate awakening proportion, activity resumption time).

### Statistical analysis

IBM SPSS Statistics 29.0 (IBM Corp, Armonk, NY, USA) and Python 3.10 (Python Software Foundation, Wilmington, DE, USA) were used for statistical analysis. Continuous variables were presented as the median with interquartile range (IQR). The Kruskal-Wallis H test or Mann-Whitney U test was utilized to assess statistically significant differences for continuous or ordinal variables. Paired comparisons of behavioural scores between dexmedetomidine and remimazolam administration in the same child were analysed with the Wilcoxon signed-rank test. Categorical data were presented as numbers and percentages, and the comparison in groups was conducted using the chi-square test or Fisher’s exact test. For the nine pre-specified secondary outcomes, a Bonferroni correction was applied to control for multiple comparisons, with statistical significance defined as *p* < 0.0056 (i.e., adjusted α = 0.05/9). Post-hoc analyses (e.g., anxiety stratification) were considered exploratory and used nominal *p*-values (*p* < 0.05).

Dose-response data were modeled using a probit regression to estimate the ED_50_ and ED_90_ of remimazolam, with precision quantified by 95% confidence intervals. The model’s goodness-of-fit was assessed by Pearson’s chi-square test (*p* > 0.05 indicating adequate fit). Isotonic regression with PAVA and bootstrapping validated the probit-derived ED_50_/ED_90_ estimates. A sensitivity analysis excluded cases with acute pain conditions exhibiting sedation failure requiring rescue propofol. Probit regression was repeated to evaluate the robustness of dose estimates.

## Results

**Fig. 1:**
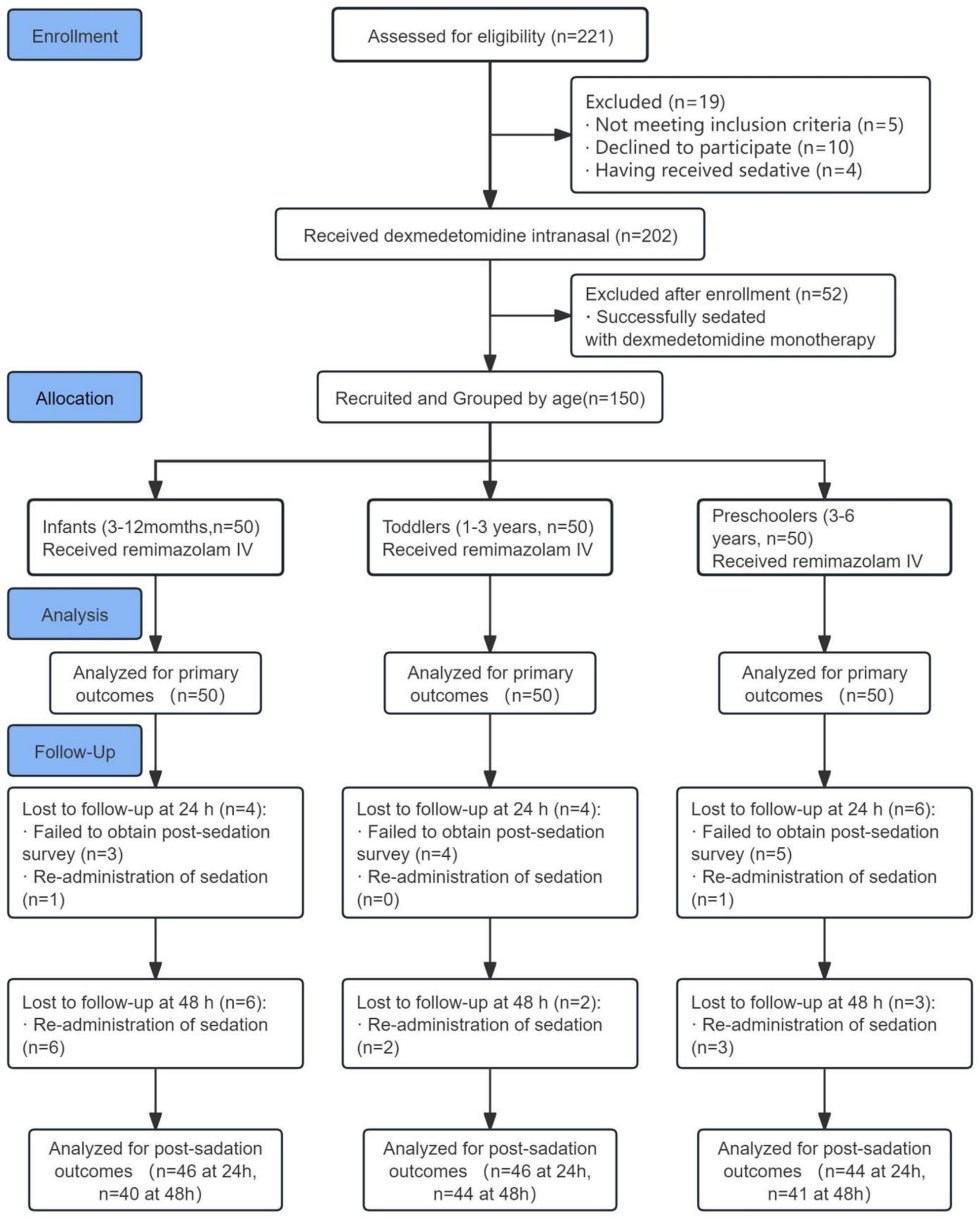
Flow diagram of the study

A total of 221 children were screened for eligibility, and 202 were enrolled. Following intranasal dexmedetomidine premedication, 52 children achieved adequate sedation and were excluded from further intervention. The remaining 150 children stratified into three age groups (50 in each group) and received intravenous remimazolam (Fig. 1).

**Fig. 2:**
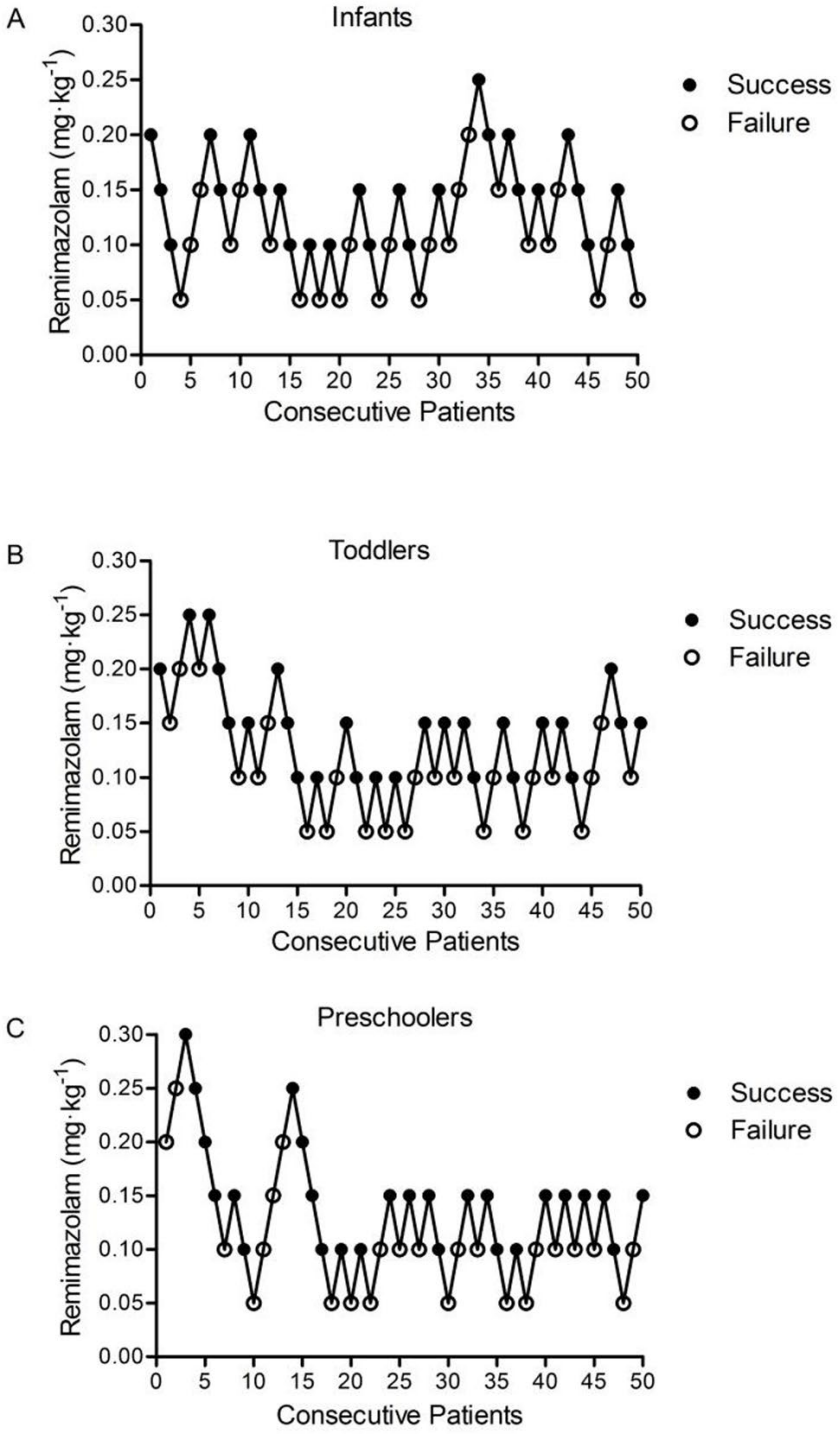
Responses of remimazolam with Dixon’s up-and-down method in each group (**A**: Infants; **B**: Toddlers; **C**: Preschoolers). The interval was 0.05 mg·kg^− 1^

Demographic data stratified by age group were presented in Table 2. Figure 2 presents the sedation outcomes for each remimazolam dose, with dose-response curves detailed in Additional file 1. The number of crossover pairs in the same direction was 14 in infants, 16 in toddlers and preschoolers. The ED_50_ and ED_90_ values of remimazolam for successful sedation were presented in Table 3 (all probit models demonstrated adequate fit with Pearson χ² *p* > 0.05). The ED_50_ values for all three age groups were 0.12 mg·kg^− 1^, while ED_90_ values increased slightly from 0.20 mg·kg^− 1^ in infants and toddlers to 0.23 mg·kg^− 1^ in preschoolers. Isotonic regression with PAVA and bootstrapping confirmed the probit-derived ED₅₀/ED₉₀ estimates (Additional file 2). Probit sensitivity analysis excluded one preschooler with acute pain (lumbar fracture, VAS: 3) who required rescue propofol. Re-analysis yielded negligible changes to dose estimates (Δ < 0.01 mg·kg⁻¹; Table 3).

**Table 2:** Demographic characteristics of patients receiving dexmedetomidine-remimazolam sequential regimen

Demographic characteristics	Infants (*n* = 50)	Toddlers (*n* = 50)	Preschoolers (*n* = 50)
Sex, male	34 (68%)	24 (48%)	32 (64%)
Age (month)	6.5 (4.0–9.0)	25.0 (16.0–31.0)	55.5 (45.0–70.0)
Weight (kg)	8.0 (7.0–9.0)	11.0 (9.8–13.5)	16.0 (14.0–18.0)
ASA			
Ⅰ	15 (30%)	23 (54%)	27 (54%)
Ⅱ	34 (68%)	26 (52%)	23 (46%)
Ⅲ	1 (2%)	1 (2%)	0
History of surgery	16 (32%)	16 (32%)	16 (32%)
Neurological comorbidity	33 (66%)	29 (58%)	34 (68%)
History of sedation	30 (60%)	34 (68%)	34 (68%)
History of sedation difficulties	2 (4%)	12 (24%)	3 (6%)
Duration of sleep deprivation (h)	3.0 (1.0–4.0)	4.0 (3.0–5.0)	5.0 (3.0–6.0)

Values in median (IQR) or number (proportion) as appropriate

**Table 3:** Effective doses and model goodness-of-fit by probit regression analysis

	Infants (*n* = 50)	Toddlers (*n* = 50)	Preschoolers (*n* = 50)
ED_50_ (mg·kg^− 1^)	0.12 (0.09–0.15)	0.12 (0.09–0.15)	0.12 (0.08–0.16)
ED_90_ (mg·kg^− 1^)	0.20 (0.16–0.29)	0.20 (0.17–0.31)	0.23 (0.18–0.42)^*^
Goodness-of-fit			
Pearson χ²	48.45	50.73	55.80
*p* Value	0.455	0.366	0.205

Values represent ED_50_ or ED_90_ with 95%CI. ED_50_, 50% effective dose; ED_90_, 90% effective dose. The Pearson goodness-of-fit chi-square test was used to assess the probit regression model. A *p* value > 0.05 indicates an adequate model fit. ^*^Sensitivity analysis excluding one preschooler with acute pain (lumbar fracture) due to sedation failure yielded ED_90_ of 0.23 mg·kg⁻¹ (95%CI 0.18–0.45). ED_50_ remained 0.12 mg·kg⁻¹ (95%CI 0.07–0.16)

The sequential sedation protocol achieved a 93.3% (140/150) success rate for MRI completion without supplemental medication during the scanning. Secondary outcomes were summarized in Table 4, with no significant differences observed among the three age groups. Among the ten cases (6.7%) requiring supplementary remimazolam during MRI, nine received a single dose and one needed multiple doses, with the first top-up administered at a median of 20.5 min (IQR: 15–35) after induction. All cases requiring top-ups involved temporary scanner pauses. These children had significantly longer examination time (median: 30 min, IQR: 23–40) compared to those without supplementation (median: 16 min, IQR: 14–22; *p* < 0.001). Similarly, awake time was prolonged in children requiring supplemental doses (median: 42 min, IQR: 35–52) versus those who did not (median: 23 min, IQR: 18–30; *p* < 0.001). Only one case (0.7%) required propofol rescue prior to MRI initiation. A total of 62 of 150 (41.3%) patients woke up immediately after the examination.

**Table 4:** Secondary outcomes of patients receiving dexmedetomidine-remimazolam sequential regimen

Outcomes	Infants (*n* = 50)	Toddlers (*n* = 50)	Preschoolers (*n* = 50)	*p* Value
Behaviour with nasal dexmedetomidine administration				
1	4 (8%)	7 (14%)	7 (14%)	0.753
2	15 (30%)	9 (18%)	13 (26%)	
3	12 (24%)	10 (20%)	9 (18%)	
4	19 (38%)	24 (48%)	21 (42%)	
Behaviour with venous remimazolam administration				
1	32 (64%)	26 (52%)	29 (58%)	0.350
2	8 (16%)	10 (20%)	15 (30%)	
3	8 (16%)	8 (16%)	3 (6%)	
4	2 (4%)	6 (12%)	3 (6%)	
Cumulative pre-MRI remimazolam dose (mg·kg^− 1^)	0.15 (0.15–0.20)	0.18 (0.15–0.25)	0.15 (0.15–0.20)	0.916
Children requiring remimazolam during MRI	5 (10%)	2 (4%)	3 (6%)	0.605
Examination time (min)	15.5 (15.0–22.0)	17.5 (14.0–24.0)	16.5 (14.0–22.0)	0.634
Awake time (min)	25.5 (19.0–31.0)	26.0 (19.0–32.0)	22.0 (18.0–30.0)	0.446
Children wake up immediately	17 (34%)	21 (42%)	24 (48%)	0.362
Time to resume age-appropriate activities (h)	3.0 (2.0−3.5)	3.0 (2.0–4.0)	2.5 (2.0–4.0)	0.255
Overall adverse events	5 (10%)	10 (20%)	8 (16%)	0.377

Values in median (IQR) or number (proportion). A *p* value < 0.0056 was considered statistically significant

In post hoc analyses, children were stratified by procedural anxiety (low: behavioral scores during dexmedetomidine administration ≤ 2; high: scores 3–4), all age strata showed a consistent pattern of greater median cumulative pre-MRI remimazolam doses in high-anxiety versus low-anxiety groups (0.20 vs. 0.15 mg·kg⁻¹), although these differences were not statistically significant (Mann-Whitney U tests, all *p* > 0.05). Additional file 3 details remimazolam dose requirements stratified by anxiety levels, with effect sizes and 95% CIs. Given that remimazolam was administered later than dexmedetomidine, behaviour scores for intravenous administration were significantly lower than those for intranasal administration in children (*p* < 0.001).

The study recorded no incidence of serious adverse events. Table 5 presents the incidence of adverse reactions during and after sedation. Adverse events occurred in 23 (15.3%) children. Hypotension (SBP ≥ 20% below age-specific norms) occurred in 2% (3/150) of children, and bradycardia (HR ≥ 20% below age-specific norms) was observed in 0.7% (1/150). Both conditions were transient and self-limiting, requiring no medical intervention. Only one patient with a recent history of bronchitis had hypoxia after intravenous administration, with a minimum oxygen saturation of 90%. The patient’s SpO_2_ returned to normal within 1 min without oxygen supplementation. The post-sedation survey was available for 136/150 (90.7%) and 125/150 (83.3%) children at 24 h and 48 h, respectively. Delayed nausea/vomiting (5.1%, 7/136) and post-sedation agitation (7.4%, 10/136) were reported, resolving in 85.7% (6/7) and 80% (8/10) within 24 h, respectively; all resolved by 48 h. No differences were found in adverse event incidence or time to resume age-appropriate activities among the three age groups. No one showed respiratory depression in the first 48 h after discharge.

**Table 5:** Adverse events of patients during and after sedation

Adverse events	During sedation (*n* = 150)	24 h after sedation (*n* = 136)	48 h after sedation (*n* = 125)
Hypotension	3 (2%)	0	0
Bradycardia	1 (0.7%)	0	0
Hypoxia	1 (0.7%)	0	0
Injection pain	2 (1.3%)	0	0
Hiccups	1 (0.7%)	1 (0.7%)	0
Nausea and vomiting	0	7 (5.1%)	1 (0.8%)
Agitation	0	10 (7.4%)	2 (1.6%)

Values in number (%)

## Discussion

This study established for the first time the ED_50_ and ED_90_ of intravenous remimazolam for sedation following dexmedetomidine premedication in paediatric MRI. The ED_50_ remained consistent across age groups, while ED_90_ values showed minimal variation, supporting weight-based dosing.

Optimal medication selection is essential for safe and effective procedural sedation during paediatric MRI. To this end, we designed a two-step protocol combining intranasal dexmedetomidine premedication with intravenous remimazolam, determining ED_50_ and ED_90_ values for remimazolam across three critical paediatric subgroups: infants (3–12 months), toddlers (1–3 years), and preschoolers (3–6 years). This age range encompasses children with developmental sedation challenges, while encompassing the critical period of rapid pharmacokinetic maturation. Previous studies on effective doses of remimazolam, even those stratified by age, often excluded infants [14, 17], despite the first year of life being a period of rapid physiological maturation that can significantly influence drug responses. Our study provided the first remimazolam dosing in infants, addressing a critical gap in the literature across this vulnerable developmental window.

Notably, 93.3% of children completed the procedure without supplementary medication during the examination. Historical reports describe success rates of 86.7% with chloral hydrate (ages 0–10 years) [23], 59% with midazolam (1–7 years) [24], 30% with intranasal dexmedetomidine alone (1–10 years) [9]. Compared to intravenous propofol infusion in similar age groups (98% success, 48/49) requiring continuous titration (interruptions: 0.22 ± 0.42) or dexmedetomidine infusion (67.4% success, 31/46) with higher interruption rates (0.81 ± 1.06) [25], our protocol achieved similar efficacy with a single remimazolam bolus. This simplified approach may enhance clinical practicality by eliminating the need for continuous infusion, particularly in resource-limited settings.

Despite developmental differences, the ED_50_ values for all three age groups were 0.12 mg·kg^− 1^, indicating that a weight-based uniform dosing strategy could be effective for paediatric sedation. The consistency in ED_50_ values may be attributed to remimazolam’s esterase-mediated metabolism, which demonstrates weight-proportional pharmacokinetics with high clearance and short context-sensitive half-time, similar to adults [15]. Our ED_50_ values were lower than those reported for remimazolam monotherapy in comparable populations: 0.19 mg·kg^− 1^ in children aged 3–15 years [26], and 0.41–0.42 mg·kg^− 1^ in toddlers and preschoolers [15]. This difference may be attributed to the potential pharmacodynamic interaction between dexmedetomidine and remimazolam, which could enhance sedative efficacy. Notably, although a slight increase in ED_90_ values was observed from 0.20 mg·kg^− 1^ in infants to 0.23 mg·kg^− 1^ in preschoolers, the overlapping confidence intervals suggested limited clinical relevance. Particularly in preschoolers, the wider confidence intervals further emphasize the need for dose titration.

To determine this effective dose, we employed the Dixon ‘up-and-down’ method, a widely recognized technique in anaesthesia studies [27]. According to Assaf’s recommendation [21], we enrolled 50 patients per group to meet the minimum requirement for ED_90_ determination and ensure precision across age subgroups. Pharmacokinetic evidence informed our protocol design: intranasal dexmedetomidine (2–3 µg·kg^− 1^) reaches peak plasma concentration at a median of 37 min (IQR 30–45) [20], while remimazolam achieves onset within 1–3 min. By timing remimazolam administration at this 30-minute window, we aimed to align dexmedetomidine’s peak effect with remimazolam’s rapid onset, potentially enabling complementary pharmacodynamic actions. This temporal coordination may contribute to the observed reduction in remimazolam dose requirements compared to monotherapy regimens. The 2-minute window in our composite endpoint was selected to standardize initial sedation assessment, balancing practicality with early stability assessment. By requiring both successful initial sedation and full scan completion without additional doses, this composite endpoint accounts for variable MRI durations and reflects real-world requirements.

The post hoc analysis indicated that children exhibiting procedural anxiety (behavioral scores 3–4) tended to require higher remimazolam doses. The observed trend, though not significant, highlights the need for behavioral evaluation in sedation planning. Weight-based dosing remains the primary standard for initial administration; however, clinicians should consider behavioral assessment to guide real-time dose adjustments in distressed children. Notably, one child with lumbar fracture required propofol rescue despite adequate analgesia. While this highlights potential sedation challenges in acute injury, its exclusion from probit regression did not alter ED_50_/ED_90_ point estimates (Δ < 0.01 mg·kg⁻¹), affirming that our dose recommendations remain valid for routine paediatric MRI.

Notably, patients requiring intraprocedural remimazolam supplementation exhibited prolonged examination times, potentially influenced by procedural complexity. The IQR distributions revealed that 75% of successful single-bolus cases completed MRI within 22 min, a clinically practical threshold aligned with the median time to first top-up (20.5 min). While these observations suggest a 20-minute threshold may help anticipate supplemental needs, our design of variable initial doses prevent definitive conclusions, warranting future studies with standardized regimens to validate this boundary and clarify dose-duration relationships.

The high procedural success and rapid recovery observed in our study are noteworthy. Compared to Li et al.’s protocol [28], which used buccal midazolam with intranasal dexmedetomidine, our combination showed a numerically shorter median awake time (22–26 min vs. 45 min). This clinical advantage may be attributed to the complementary pharmacodynamic profiles of dexmedetomidine and remimazolam, which we hypothesize act synergistically rather than additively. Dexmedetomidine, an α2-adrenoceptor agonist, suppresses locus coeruleus noradrenaline release, inducing a natural sleep-like state, while remimazolam potentiates γ-aminobutyric acid (GABA)-mediated chloride influx through GABAA receptor activation [29]. This potential synergy, acting on distinct noradrenergic and GABAergic pathways, is supported by preclinical evidence demonstrating enhanced sedative effects when dexmedetomidine is combined with the benzodiazepine midazolam [30]. Meanwhile, dexmedetomidine’s activation of ventral tegmental area dopamine neurons potentially preserves arousability [31], which may explain why 41.3% of children awakened immediately post-MRI despite profound procedural immobility. Remimazolam’s esterase-mediated hydrolysis further ensures predictable clearance, offering advantages over traditional agents with improved cardiorespiratory stability.

Our study observed low incidences of bradycardia (0.7%) and hypoxia (0.7%), with hypotension occurring in 2% of cases. In comparison, a large retrospective propofol study reported bradycardia in 2.67% and hypoxia in 4.21% [2], while a propofol-dexmedetomidine combination showed 10.9% bradycardia and 3.6% upper airway obstruction [32]. Fang et al.’s RCT on remimazolam for paediatric anaesthesia reported 9% of bradycardia rates [18], possibly reflecting distinct procedural contexts. The higher incidence of post-sedation nausea (5.1%) and agitation (7.4%) should be interpreted cautiously, as guardian-reported outcomes may be influenced by recall bias and underlying disease-related discomfort. Our protocol demonstrated a numerically shorter time to resume age-appropriate activities compared to dexmedetomidine-midazolam regimens (median 2.5–3.0 h vs. 5.5 h) [28], a difference that may reflect remimazolam’s esterase-based metabolism and shorter context-sensitive half-time. These preliminary findings suggest that our sedation protocol may offer practical advantages, though randomized controlled trials are needed to validate comparative efficacy and safety.

### Limitations

This study has several limitations. Firstly, as a single-center investigation specifically enrolling dexmedetomidine-non-responders aged 3 months to 6 years, the results are most directly applicable to this defined subgroup within similar clinical settings. While the single-arm design is appropriate for dose-finding, it precludes direct evaluation of the assumed synergy between dexmedetomidine and remimazolam. The pragmatic inclusion of children with neurological comorbidities, while reflecting clinical practice, may influence sedative response. Consequently, extrapolation to all paediatric MRI patients, particularly those with significant comorbidities or from different demographic groups, requires caution and validation.

Sedation depth was assessed using the validated but inherently subjective UMSS. Our composite endpoint, though clinically holistic, precludes separate evaluation of distinct induction and maintenance requirements. Preoperative anxiety was assessed using a pragmatic behavioral scale rather than a standardized instrument, and pain was not systematically evaluated. Our analysis did not adjust for these factors or procedural duration; any post-hoc exploration is underpowered. The absence of end-tidal carbon dioxide (EtCO₂) and processed electroencephalogram monitoring limited objective quantification of hypoventilation or excessive sedation depth, potentially underestimating subclinical adverse events. Partial gaps in vital-signs data before sedation and upon awakening further constrained safety profiling. Furthermore, parental reporting bias in post-discharge surveys, while common in paediatric studies, remains unavoidable.

While the sample size satisfied minimum requirements for ED_90_ estimation, the study may not be powered for probit regression of dependent data or rare adverse event detection. The wide confidence interval for ED_90_ in preschool children suggests that larger cohorts would strengthen dosing precision. The absence of plasma concentration measurements limits pharmacokinetic characterisation, and the variable bioavailability of intranasal dexmedetomidine may contribute to dose variability.

To enhance generalizability, a randomized controlled trial (RCT) is underway. However, broader adoption of remimazolam may be limited by its variable regulatory status and higher cost compared to agents like propofol. Future multicentre trials with larger, more diverse cohorts should incorporate pharmacoeconomic analyses and pursue regulatory harmonization to enhance the external applicability.

## Conclusions

In conclusion, our study defines the ED_50_ (0.12 mg·kg^− 1^) and ED_90_ (0.20–0.23 mg·kg^− 1^) of remimazolam for sedation after dexmedetomidine premedication in children aged 3 months-6 years who were dexmedetomidine-non-responders undergoing MRI. The consistent ED_50_ across age groups supports weight-based dosing, while the border ED_90_ confidence intervals highlight the need for individualized titration. Given its high success rate with a favourable haemodynamic and respiratory safety profile, this sequential regimen warrants further investigation.

## Supplementary Information

Additional file 1: Dose-response relationship of remimazolam for successful sedation in each group (A: Infants; B: Toddlers; C: Preschoolers). The black line represents the typical value of the model simulation, with the grey shaded area representing the 95% confidence interval of the typical value simulated by the model.

Additional file 2: Effective doses of remimazolam in age groups by isotonic regression analysis. Values represent ED_50_ or ED_90_ with 95%CI. ED_50_, 50% effective dose; ED_90_, 90% effective dose.

Additional file 3: Post hoc analysis of cumulative pre-MRI remimazolam requirements stratified by anxiety level. Remimazolam requirements were compared between high- and low-anxiety groups within each age stratum using the Mann-Whitney U test. A positive effect size (r) indicates higher doses in the high-anxiety group. A *p* value < 0.05 was considered statistically significant.

## Data Availability

Requests for access to deidentified participant data may be directed to the corresponding author.

## Abbreviations

MRI: Magnetic resonance imaging
ED_50_: 50% effective dose
ED_90_: 90% effective dose
ASA: American Society of Anesthesiologists
OSA: Obstructive sleep apnoea syndrome
UMSS: University of Michigan Sedation Scale
PR: Pulse rate
NBP: Non-invasive blood pressure
SpO_2_: Oxygen saturation
WHO: World Health Organization
SBP: Systolic blood pressure
IQR: Interquartile range
PAVA: Pooled-adjacent-violators algorithm
VAS: Visual Analog Scale
GABA: γ-aminobutyric acid
GABAA: γ-aminobutyric acid type A receptor
EtCO₂: End-tidal carbon dioxide
RCT: Randomized controlled trial
