# Supplementary figures and images for: Effective doses of remimazolam for sedation in paediatric magnetic resonance imaging following dexmedetomidine premedication: a dose-finding study
Source: BMC Anesthesiol. 2026 Apr 20;26:338. doi: 10.1186/s12871-026-03848-2 (PMC13224665; doi:10.1186/s12871-026-03848-2)

Infants

A

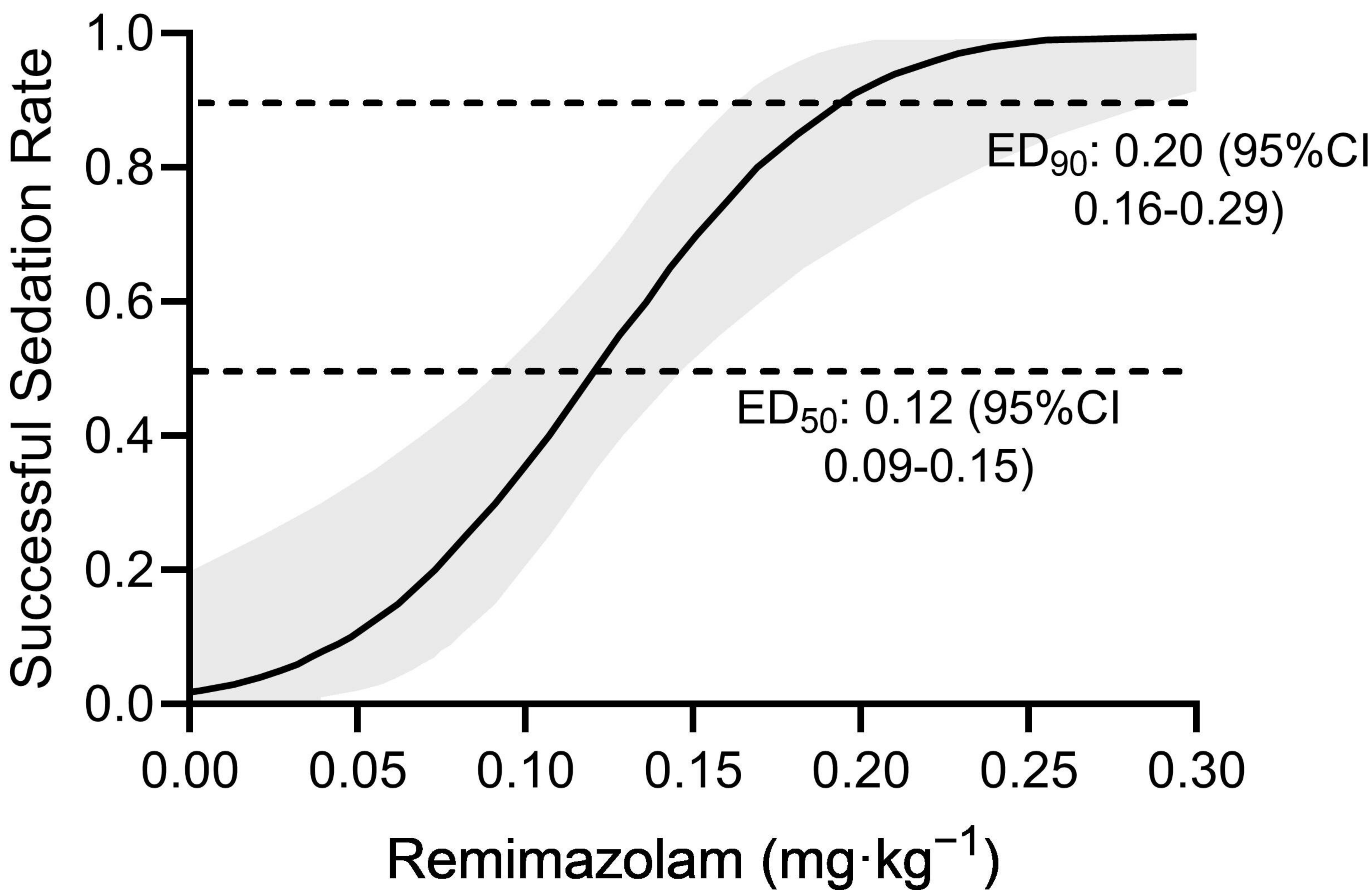

Toddlers

B

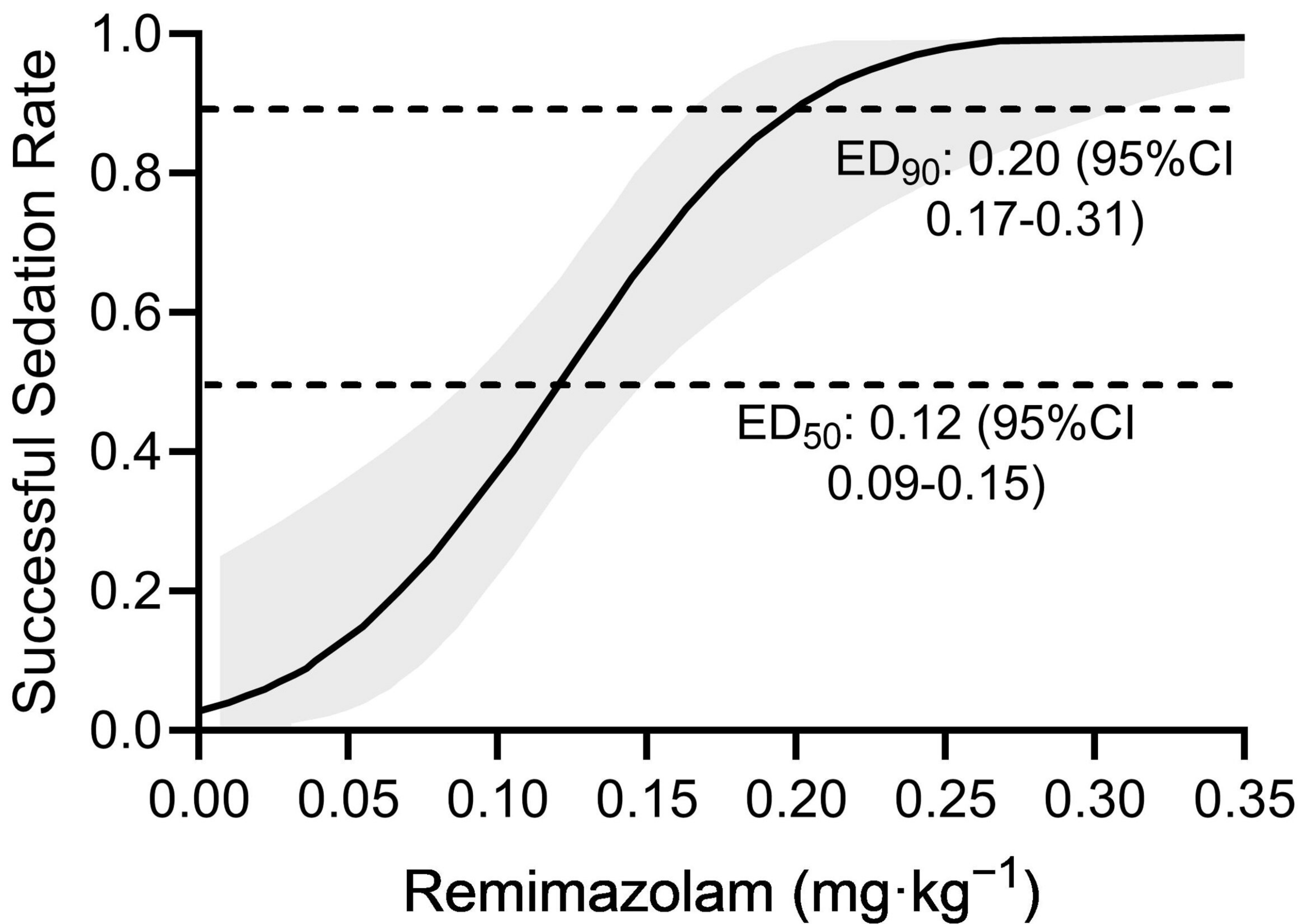

Preschoolers

C

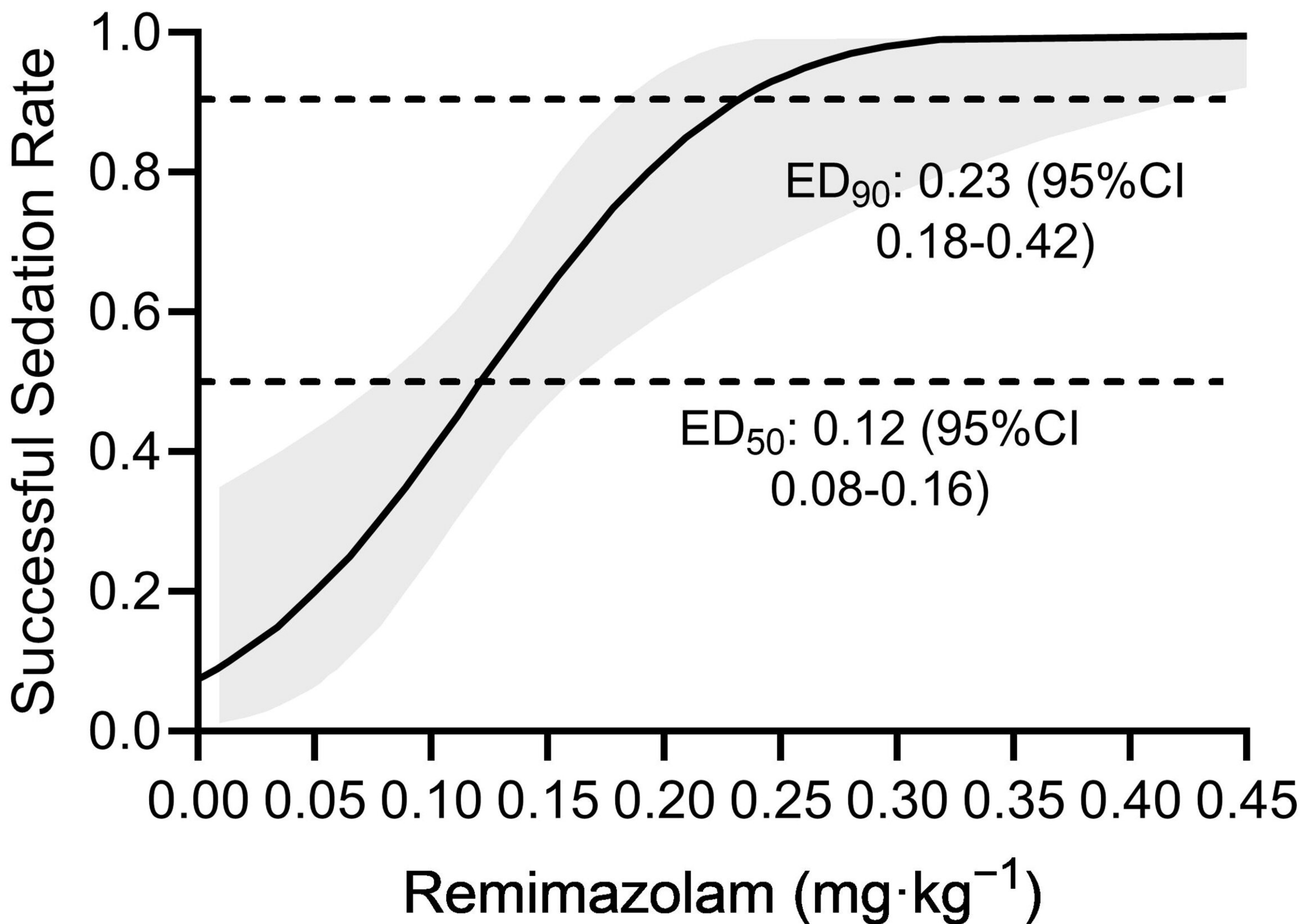

Supplement: Supplementary file 1 — Additional file 1: Dose-response relationship of remimazolam for successful sedation in each group (A: Infants; B: Toddlers; C: Preschoolers). The black line represents the typical value of the model simulation, with the grey shaded area representing the 95% confidence interval of the typical value simulated by the model. [file 12871_2026_3848_MOESM1_ESM.pdf]
