# Supplementary material for: Effective doses of remimazolam for sedation in paediatric magnetic resonance imaging following dexmedetomidine premedication: a dose-finding study
Source: BMC Anesthesiol. 2026 Apr 20;26:338. doi: 10.1186/s12871-026-03848-2 (PMC13224665; doi:10.1186/s12871-026-03848-2)
Supplement: Supplementary file 2 — Additional file 2: Effective doses of remimazolam in age groups by isotonic regression analysis. Values represent ED50 or ED90 with 95%CI. ED50, 50% effective dose; ED90, 90% effective dose. [file 12871_2026_3848_MOESM2_ESM.pdf]

Additional file 2 Effective doses of remimazolam in age groups by isotonic regression analysis

	Infants (n=50)	Toddlers (n=50)	Preschoolers (n=50)
ED_{50} (mg·kg^{-1})	0.11 (0.09-0.16)	0.11 (0.09-0.14)	0.11 (0.09-0.13)
ED_{90} (mg·kg^{-1})	0.22 (0.16-0.24)	0.23 (0.15-0.24)	0.27 (0.15-0.29)

Values represent ED_{50} or ED_{90} with 95%CI. ED_{50}, 50% effective dose; ED_{90}, 90% effective dose.
