# Supplementary material for: Effective doses of remimazolam for sedation in paediatric magnetic resonance imaging following dexmedetomidine premedication: a dose-finding study
Source: BMC Anesthesiol. 2026 Apr 20;26:338. doi: 10.1186/s12871-026-03848-2 (PMC13224665; doi:10.1186/s12871-026-03848-2)
Supplement: Supplementary file 3 — Additional file 3: Post hoc analysis of cumulative pre-MRI remimazolam requirements stratified by anxiety level. Remimazolam requirements were compared between high- and low-anxiety groups within each age stratum using the Mann-Whitney U test. A positive effect size (r) indicates higher doses in the high-anxiety group. A p value < 0.05 was considered statistically significant. [file 12871_2026_3848_MOESM3_ESM.pdf]

Additional file 3 Post hoc analysis of cumulative pre-MRI remimazolam requirements stratified by anxiety level

Age group	Anxiety group	Cumulative pre-MRI remimazolam(mg·kg^{-1})	Effect size r (95%CI)	*p* Value
Infants (n=50)	Low-anxiety (n=19)	0.15 (0.13-0.20）	0.243 (-0.034-0.52)	0.086
	High-anxiety (n=31)	0.20 (0.15-0.20）		
Toddlers (n=50)	Low-anxiety (n=16)	0.15 (0.10-0.20）	0.196 (-0.081-0.473)	0.166
	High-anxiety (n=34)	0.20 (0.15-0.25）		
Preschoolers (n=50)	Low-anxiety (n=20)	0.15 (0.15-0.20）	0.150 (-0.127-0.427)	0.290
	High-anxiety (n=30)	0.20 (0.15-0.25）		

Data are presented as median (IQR) for cumulative remimazolam dose. Remimazolam requirements were compared between high- and low-anxiety groups within each age stratum using the Mann-Whitney U test. A positive effect size (r) indicates higher doses in the high-anxiety group. A p value < 0.05 was considered statistically significant.
